# Gluten-Free Diet and Other Celiac Disease Therapies: Current Understanding and Emerging Strategies

**DOI:** 10.3390/nu16071006

**Published:** 2024-03-29

**Authors:** Anna Maria Mazzola, Irene Zammarchi, Maria Chiara Valerii, Enzo Spisni, Ilaria Maria Saracino, Francesco Lanzarotto, Chiara Ricci

**Affiliations:** 1 Gastroenterology Unit, Spedali Civili Hospital, 25123 Brescia, Italy; annamaria.mazzola01@universitadipavia.it (A.M.M.); irene.zammarchi01@universitadipavia.it (I.Z.); francesco.lanzarotto@asst-spedalicivili.it (F.L.); 2 Department of Internal Medicine and Medical Therapy, University of Pavia, 27100 Pavia, Italy; 3 Unit of Translational Physiology and Nutrition, Department of Biological, Geological and Environmental Sciences, University of Bologna, 40126 Bologna, Italy; mariachiara.valerii2@unibo.it (M.C.V.); enzo.spisni@unibo.it (E.S.); 4 Microbiology Unit, IRCCS, Azienda Ospedaliero-Universitaria di Bologna, University of Bologna, Via Massarenti 9, 40138 Bologna, Italy; ilariamaria.saracino@studio.unibo.it; 5 Department of Experimental and Clinical Science, University of Brescia, 25123 Brescia, Italy

**Keywords:** adherence, contamination, gluten-free diet, nutrition, psychological aspects

## Abstract

A lifelong gluten-free diet (GFD) is the only treatment for celiac disease and other gluten-related disorders. Nevertheless, strict adherence to the GFD is often challenging due to concerns about social isolation, risk of gluten contaminations, high cost, poor quality and the taste of gluten-free products. Moreover, although the GFD is effective in achieving mucosal healing, it may lead to dietary imbalances due to nutrient deficiencies over a long period of time. To overcome these issues, several gluten-free wheat flours have been developed to create products that closely resemble their gluten-containing counterparts. Furthermore, given the critical importance of adhering to the GFD, it becomes essential to promote adherence and monitor possible voluntary or involuntary transgressions. Various methods, including clinical assessment, questionnaires, serology for celiac disease, duodenal biopsies and the detection of Gluten Immunogenic Peptides (GIPs) are employed for this purpose, but none are considered entirely satisfactory. Since adherence to the GFD poses challenges, alternative therapies should be implemented in the coming years to improve treatment efficacy and the quality of life of patients with celiac disease. The aim of this narrative review is to explore current knowledge of the GFD and investigate its future perspectives, focusing on technology advancements, follow-up strategies and insights into a rapidly changing future.

## 1. Introduction

Gluten-related disorders can be classified into three categories according to their pathogenesis: autoimmune (celiac disease [CD]), allergic (IgE- or non-IgE-mediated wheat allergy [WA]) and non-autoimmune/non-allergic (non-celiac gluten sensitivity [NGCS]) [1]. CD is an autoimmune disease in which genetic factors (HLA-DQ2 and HLA-DQ8), auto-antibodies (anti-transglutaminase) and environmental factors (gluten ingestion) contribute to the development of the disease, resulting in intestinal (diarrhoea, bloating and constipation) and extraintestinal (weight loss, anaemia, osteoporosis, dermatitis herpetiformis and neurological disorders) manifestations. When gluten is ingested, the gliadin fragments bind to the chemokine receptors on the apical side of enterocytes, causing the release of zonulin, which increases intestinal permeability and the translocation of gliadin fragments to the lamina propria, the consequent production of IL-8 and IL-15 and the recruitment of neutrophils. The apoptosis of intestinal cells stimulated by the innate immunity causes the release of tissue transglutaminase (TG2) with partial deamination of gliadin fragments in 33-mer fragments, which are recognised by DQ2 and DQ8 antigen-presenting cells. All these events lead to T helper cell activation and B cell maturation, with the production of IgA, IgM and IgG antibodies against tissue transglutaminase, responsible for intestinal damage [2]. Gluten-containing grains are rye, barley, spelt and wheat, including ancient varieties such as einkorn and kamut [3]. Oat is a cereal which is naturally gluten-free, but once on the market it can be contaminated by gluten-containing cereals at various stages of cultivation or food production [4].

CD prevalence is estimated to be around 1% globally, but it appears to be growing, with a patchy distribution and regions reaching up to 3% (sero-prevalence) in Saudi Arabia [5,6]. Moreover, it often goes undiagnosed [7]. Genetic predisposition plays a crucial role in the development of the disease, particularly the presence of HLA-DQ2 and HLA-DQ8 alleles, but factors related to the environment also seem to have an important effect, responsible especially for the increase in prevalence observed in some areas of the world. As a result of an immune response to gluten ingestion, CD is primarily characterized by small-intestinal mucosal damage, and can have a wide range of clinical presentation, from asymptomatic forms to intestinal and extraintestinal symptoms [8].

WA is a hypersensitivity reaction to wheat proteins that results in an allergic response upon contact, inhalation, or the consumption of foods containing wheat, although not necessarily to other grains like barley or rye. However, some individuals may exhibit cross-reactivity to other gluten-containing cereals [9,10,11]. Even if the molecular mechanisms underlying WA have not been completely explained, they share similarities to other food allergies [11].

Non-celiac gluten sensitivity is a condition in which patients develop symptoms like CD or WA (abdominal pain, distention, bloating, diarrhoea, fatigue, headaches, etc.) hours or days after the consumption of wheat, in the absence of IgA anti-tTG or IgE against wheat. The pathogenesis of NCGS is still unknown [12].

Currently, a gluten-free diet (GFD) is the cornerstone of management of CD. For other gluten-related disorders, the diet may be different and contain certain cereals with gluten but not wheat (in the case of WA) or limit only the quantities of gluten (in the case of NCGS, in which small doses of gluten or contamination generally do not cause symptoms). In these cases, the level of dietary strictness depends on the specific clinical condition.

Progress has been made in the development of gluten-free wheat flours. Evaluating the quality and safety of these products is crucial to addressing nutritional deficiencies and to preventing gluten contamination, respectively [13,14]. The introduction of new gluten-free flours offers an alternative for individuals seeking gluten-free options while maintaining the sensory and nutritional qualities of traditional wheat-based products.

However, adhering to a GFD can have a significant impact on the psychosocial quality of life of patients. Individuals on a gluten-free diet have to manage psychosocial challenges, including dietary restrictions, social limitations and the emotional impact of managing a lifelong condition [15]. For these reasons, several methods have been developed to evaluate the adherence to a GFD for CD patients, to prevent potential harmful consequences caused by incorrect dietary practices [16]. Moreover, strategies and interventions that can help improve the psychosocial well-being of these patients are required. Although the need for a GFD is widely acknowledged, its restrictions have prompted the exploration of novel therapeutic options that seem to hold promise for reducing the impact of the diet and thereby enhancing patients’ quality of life. Recent studies seem to focus on two approaches: improving the quality of gluten-free products by using new ingredients and flours, and abandoning the idea of the absence of gluten traces, which requires obsessive attention to product certification and to the choice of restaurants, is poorly accepted by patients and, finally, is almost impossible to achieve.

The aim of this narrative review is to provide an overview of current knowledge on GFDs, the safety of new gluten-free wheat flours and potential future treatments for celiac patients. Furthermore, we analyse here possible new methods for monitoring adherence to a GFD diet, looking ahead to the future and the fast-moving advancements in research and food technologies.

## 2. Materials and Methods

**Figure 1:**
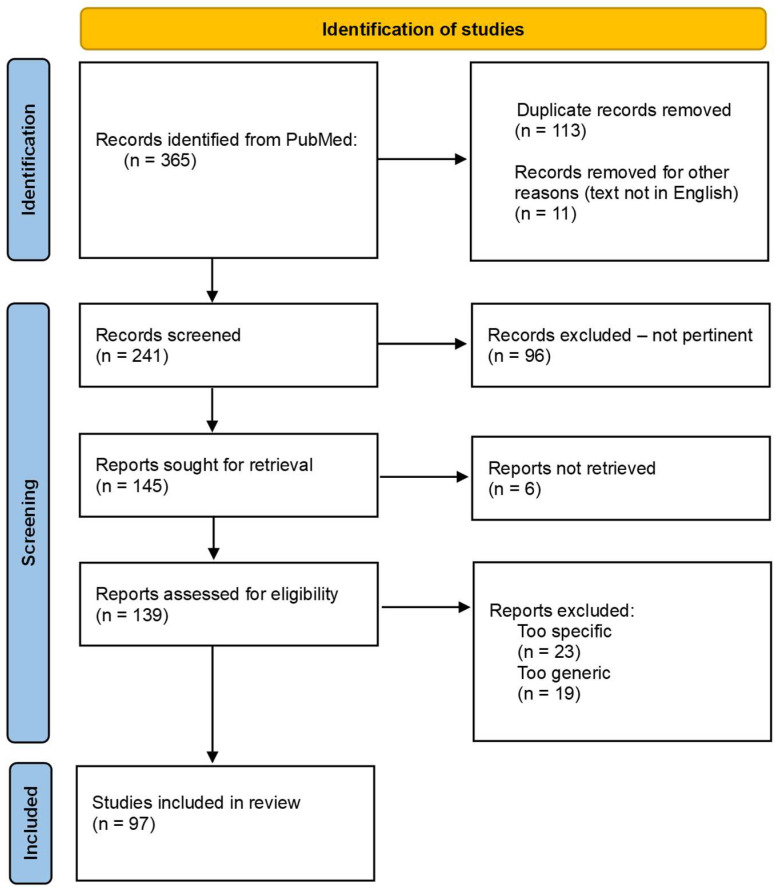
PRISMA flow diagram.

A literature review was conducted on databases, including PubMed, with “advanced” and “MeSH” tools, using the following key term queries: (celiac disease AND gluten-free diet) [title]; ((gluten-related disorder*) OR ((gluten-related disease*) AND (quality of life))) [title/abstract]; “gluten-free wheat flour” [title/abstract]; ((adherence to) OR (challenges of) AND gluten free diet“) [title/abstract]; “gluten contamination” [title/abstract]; “Gluten Immunogenic Peptides detection” [all fields]. Inclusion criteria were as follows: narrative and systematic reviews, clinical trials, meta-analyses and randomized controlled trials, published in the last 10 years, giving priority to the most recent. Exclusion criteria were as follows: clinical case studies, letters or editorials of magazines, abstracts to conferences, book chapters and unpublished materials. PRISMA flow diagram is reported in Figure 1.

A detailed bibliographic search was also carried out in the references of the selected articles to identify other studies that might be useful for the purpose of the review.

## 3. Results

The results cover six main areas: (1) appropriate use of a GFD; (2) adherence to the GFD and how to monitor it; (3) safety of gluten-free labelled and unlabelled foods (risks of contaminations); (4) nutritional imbalances related to the GFD; (5) assessment of the psychosocial quality of life in patients on the GFD; and (6) current and future prospects of the GFD.

### 3.1. Gluten-Free Diet: When to Use?

Strict adherence to a GFD is the primary effective treatment for managing symptoms and preventing complications associated with CD. Gluten exclusion from the diet is the only available treatment for CD and it usually results in an improvement in clinical manifestations, as well as the normalization of serological markers [17] and mucosal healing. Within two years, 95% of children achieve a mucosal recovery, while only 34% and 66% of adults obtain it in two and five years, respectively [18,19,20]. However, a complete mucosal recovery is rare, even with a carefully followed GFD: Marsh I and II lesions persist in 65% of patients with atrophy at diagnosis after 6–18 months of GFD, and the Marsh 0 stage is seen in only 8% of patients [21]. Moreover, the persistence of intraepithelial lymphocytosis is independent from cross-contaminations, and it is not eliminated even when all gluten contaminations are strictly excluded from the diet [22]. Furthermore, CD patients are at increased risk of developing cancer, with a twofold [23] and a threefold [24] increased risk of non-Hodgkin lymphoma and small intestinal adenocarcinoma, respectively. Thus, a strict follow-up of these patients is crucial.

For individuals with NCGS, following a GFD is not mandatory, even if it is recommended to manage their symptoms. A GFD also appears to be useful to manage irritable bowel syndrome (IBS), as most patients refer to a correlation between the onset of symptoms and the ingestion of food containing gluten [25].

Finally, the strict avoidance of wheat and wheat-containing products is necessary for WA [26]. If WA allergens belong to the gluten protein group, patients should avoid all gluten-containing food, and strictly follow a GFD.

It is important to note that a GFD is not recommended for healthy individuals or subjects without gluten-related disorders. Despite this, up to 25% of the population in Western countries choose to adhere to a GFD, often due to fads related to weight-loss diets, without medical necessity [27]. This misconception is fuelled by ideas propagated through social media [28], suggesting that a GFD is healthier. On the other hand, it is known that diet plays a significant role in inducing inflammation, and that food rich in processed carbohydrates (almost always wheat) and saturated fats, which are the cornerstone of the so-called “Western diets”, can trigger a proinflammatory response [29]. Ongoing research is evaluating how dietary factors can influence inflammatory responses, leading to increased or reduced risk of chronic inflammation, and linked to chronic non-communicable diseases such as cardiovascular disease, obesity and cancer [30].

However, there is no evidence regarding the benefits of a GFD in the overall healthy population. Lebwohl et al. [31] suggested that a GFD may result in a reduced consumption of wheat nutrients such as fibre, B vitamins and selenium, with a consequent increase in cardiovascular risk. Moreover, several studies demonstrated an increased body weight in CD patients after the diagnosis and the beginning of the GFD [32].

In conclusion, while a strict GFD is crucial for individuals with CD and WA, adherence to the diet is not mandatory for NCGS patients; it is sometimes recommended, but sometimes it is sufficient to reduce the amounts of gluten in the diet. However, unnecessary adoption of a GFD by individuals without gluten-related disorders can lead to potential health risks and should be avoided without proper medical guidance.

### 3.2. Adherence to GFD

Maintaining a lifelong strict GFD can be extremely challenging for individuals with CD, leading to intentional or unintentional breaks in the diet. Despite the availability of GF products in developed countries increasing enormously during the last decades, their distribution is very heterogenous, and varies between regions and countries. Moreover, the cost of GF products is very high compared to analogue gluten-containing food, and national health systems only partially cover these differences [33]. As a result, people living in places where the availability of GF products is limited or in poor condition have a higher probability of breaking the diet intentionally.

Moreover, despite the significant improvements in GF food technology in recent years, many GF products are still described as having low quality, especially in terms of flavour, consistency, mouthfeel and texture. In addition, alternative raw materials that are naturally gluten-free are often not well accepted by patients [34].

Finally, label reading is another obstacle that CD patients face on a daily basis [35]. As observed by Muhammad and collaborators [35], 73% of patients who reported difficulties in understanding food labels had a higher risk of transgression when compared to 45% of patients who could correctly interpret them. Thus, understanding food composition is an important skill for CD patients to acquire to avoid unintentional gluten ingestion.

Adherence to a GFD can also be influenced by other factors such as gender, sex, age and education. Comino and coworkers [36] reported that the risk of diet transgression increases in parallel with a patient’s age, with a minimum in children under 3 years old, and a maximum in adolescents and adults over 13 years old. For CD adolescents, the adherence to the GFD becomes more difficult because of social pressure (e.g., eating out of the home with friends, fear of feeling different, etc.), with frequent occasions to break the diet [18]. Other studies suggested that the longer a patient follows the GFD, the higher the risk of non-adherence to the GFD becomes [36,37].

A recent systematic review [38] analysed eight studies (clinical trials and RCTs) in order to identify the best interventions to improve adherence to the GFD. All of these had the purpose of increasing the consciousness of patients regarding CD and the GFD, to reduce the risk of voluntary or involuntary transgressions. They involved education classes, individual training, periodical telephone calls or short message services (SMS). All interventions were effective in increasing compliance to the diet and improved quality of life and satisfaction with the diet; on the contrary, no impacts on gastrointestinal symptoms were recorded. In the era of telemedicine and social media, there is a pressing need for additional research aimed at the integration of these innovative communications channels into routine clinical practice. This integration is crucial for the purpose of enhancing patient consciousness, especially that of children and adolescents.

### 3.3. Monitoring Adherence to the GFD

Current guidelines suggest several ways to follow up with CD patients [39]. However, as the adherence rate may vary from 42% to 91% in the same cohort, depending on the method assessed [40], there is no agreement on the best tool to evaluate it. Whatever tool is used, the known advantages and disadvantages are highlighted (Table 1).

#### 3.3.1. Clinical Assessment

The amelioration of symptoms can serve as a motivating factor for adhering rigorously to the prescribed diet. Among symptomatic patients, an inadequate response to the diet has been associated with gluten consumption and enduring mucosal damage [41].

However, given the heterogeneity of CD clinical phenotypes and that more than 20% of patients are asymptomatic [2], clinical assessment alone is not sufficient to evaluate compliance to the GFD and is considered a poor predictor of mucosal healing. Moreover, symptoms can be the consequence of the coexistence of other conditions, such as IBS.

Despite these complexities, some patients asymptomatic at diagnosis report the onset of symptoms with incidental gluten ingestion during the GFD [41].

#### 3.3.2. Questionnaires

Periodic assessment performed by a professional has been demonstrated to be effective in improving compliance to the GFD [42]. Structured and validated questionnaires are a valuable tool to measure adherence to the diet, and the most frequently used are the Standardized Dietician Evaluation (SDE), the Celiac Dietary Adherence Test (CDAT) and the Biagi score. All these tools provide significant information but have the important limit of being subjective and based on the patient’s knowledge and truthfulness. Thus, they cannot identify involuntary or voluntary transgressions [34,43,44].

#### 3.3.3. Serology

Serological testing, including anti-deamidated gliadin peptide (dGp) and anti-tissue Transglutaminase (tTg) antibodies, is one of the most widely used methods of verifying dietary adherence to a GFD. A complete normalization of antibody levels can require several months or even years [44], and the persistence of elevated or fluctuating levels of antibodies can be a warning about perpetrated gluten ingestion [36]. However, serological testing cannot identify contaminations or small transgressions and does not completely reflect mucosal healing, as up to 10% of CD patients have a negative serology but villous atrophy [36]. The normalization of auto-antibody levels can be used to incentivize adherence to the diet, but the focus should be on the trend and fluctuation of antibody levels rather than their absolute value [41].

#### 3.3.4. Endoscopy

Endoscopic examination with duodenal biopsies is the most objective method for evaluating adherence, but it is invasive and expensive and there is no agreement regarding the time to perform it. Additionally, in adults, complete mucosal healing is not always possible, and it can take up to five years [21,22]. Thus, endoscopic control is suggested over this period, especially after three or four years, to avoid unnecessary controls [45]. An earlier esophagogastroduodenoscopy is suggested in patients with a high risk of an incomplete mucosal recovery, severe damage, or a persistence of symptoms despite a strict GFD and diagnosis acquired in adult life. Once mucosal healing is obtained, other endoscopies are not required, as long as the patient remains clinically asymptomatic [41].

#### 3.3.5. Detection of Gluten Immunogenic Peptides (GIPs) in Faeces and Urine

The main components of gluten are prolamins, which have a high content of proline and glutamine. Proline creates complex secondary structures which are partially resistant to hydrolysis by enzymes in the gastrointestinal tract, generating a mixture of peptides with different lengths. The principal peptide is α-gliadin, composed of 277 amino acids. Once α-gliadin reaches the lamina propria of the mucosa, it is processed by tissue transglutaminase, resulting in the production of a 25-mer (p 31–55) and a 33-mer (p 55–87) fragment. The 25-merfragment is considered toxic due to the peptide sequence p 31–43, while the 33-mer fragment is known to be immunogenic and is called a GIP. Via its sequence p 56–75 (LQLQPFPQPQLPYPQPQLPY), which has been identified as an epitope, it is presented by antigen-presenting cells to CD4+ lymphocytes, triggering the immune cascade of CD [46].

**Figure 2:**
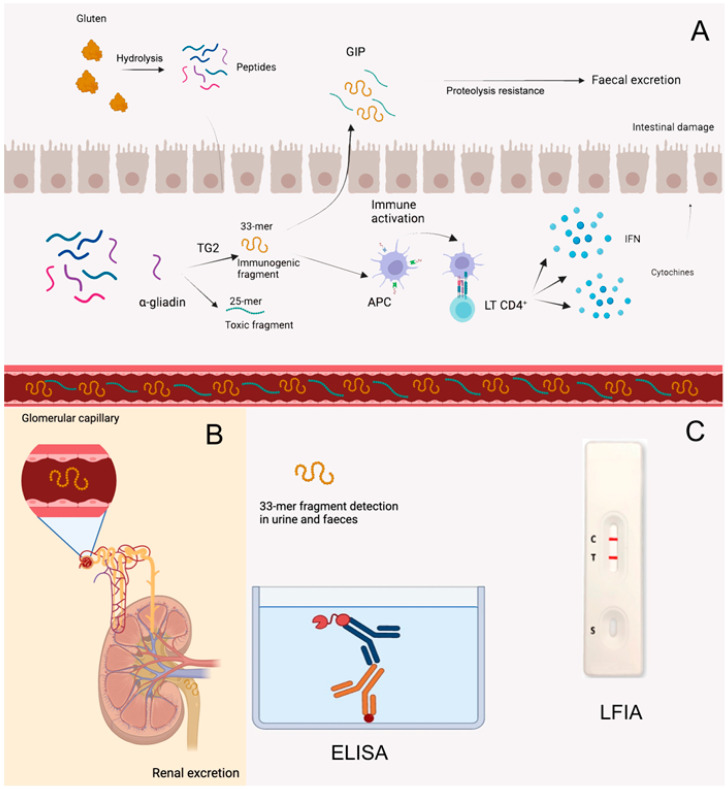
GFD-monitoring through Gluten Immunogenic Peptide (GIP) detection. (**A**) Gluten components are partially resistant to hydrolysis by enzymes in the gastrointestinal tract, generating peptides of different lengths. These peptides translocate from the lumen to the lamina propria through the damaged epithelium. Among them, α-gliadin is processed by tissue transglutaminase, with the production of a 25-mer and a 33-mer fragment, called a GIP (Gluten Immunogenic Peptide). The GIP is then presented by APCs to CD4+ lymphocytes, triggering the immune cascade of CD. (**B**) The GIP is resistant to any other rearrangement, and it can be found unmodified in faeces and urine. (**C**) Its detection can be performed with ELISA and LFIA. Abbreviations: APC: antigen-presenting cells; ELISA: enzyme-linked immunosorbent assay; GIP: Gluten Immunogenic Peptide; LFIA: lateral flow immunoassay; LT CD4+: lymphocytes CD4+; TG2: transglutaminase 2; IFNγ: interferon-γ.

The 33-mer fragment is resistant to any other rearrangement, and more than 30% of GIPs coming from ingested gluten can be found unmodified in faeces and urine (Figure 2), thanks to a detection method involving monoclonal antibodies called G12 and A1.

At the moment, the two techniques available to identify GIPs are the enzyme-linked immunosorbent assay (ELISA) and the lateral flow immunoassay (LFIA), which have similar analytical sensitivities of 0.16 µg/g and 0.15 µg/g, respectively [47].

Despite the amount of excreted GIP being hard to predict because it depends on the amount of gluten and the type of food ingested, age, sex, fluid assumed and gut microbiota metabolism, it is observed that, after gluten ingestion, GIPs are almost always positive for 6–12 h and 3–5 days in urine and faeces, respectively. The more gluten ingested, the greater the GIP excretion [48]. GIP detection is a simple, promising and cheap method to investigate adherence to the GFD.

In a study conducted in 2020 [49], the DOGGIE BAG Study Group demonstrated that transgressions on a GFD were very common; CD patients considered to have excellent compliance to the GFD, based on auto-antibody detection, had positive GIPs, confirming an unexpected gluten consumption. Moreover, two-thirds of patients with positive GIPs had villous atrophy Marsh 3a, whereas two-thirds of patients with negative GIPs had Marsh 0–1. These results suggest that there is poor correspondence between GIPs and auto-antibodies, and that GIP positivity is quite a good predictor of mucosal damage. Therefore, GIP detection can be useful to discriminate villous atrophy due to unintentional gluten ingestion and refractory CD. Similar results were obtained by Moreno and coauthors [50], who analysed the correlation between GIPs in urine and duodenal biopsies. A total of 50% of subjects on a GFD had GIP positivity in urine; 89% of patients with mucosal healing had GIP-; whereas all patients with villous atrophy had GIP+.

Finally, Porcelli and coworkers [37] evaluated the compliance of 55 CD patients on a GFD for two years using the Biagi questionnaire and GIPs in stools. A total of 8/55 patients showed GIP positivity, 94.6% of patients following a strict GFD according to the Biagi score had GIP-, and 42.9% of non-compliant patients detected by the score had GIP+. However, 57.1% of GIP+ patients were proved to be strictly adherent to the diet by the Biagi score, and GIP detection failed to recognize the 5.4% of patients who declared voluntary transgressions. As the Biagi score proved to be a good predictor of adherence to the diet, the combined use of GIP detection and the Biagi score can be the most successful way to monitor the diet.

Despite these promising results, methods for GIP detection, its fields of application and the correlation between serum antibodies and mucosal damage has still to be completely understood. For example, in contrast with the aforementioned studies, Laserna-Mandieta and coworkers [51] found a similar sensitivity (33%) and specificity (81%) between GIPs and anti-tTG antibodies in detecting mucosal damage.

#### 3.3.6. miRNAs

The most innovative diagnostic tools to emerge in recent years are urinary miRNAs, short non-coding RNAs which play a role in post-transcriptional gene expression. The first field of application for miRNAs was type 2 diabetic nephropathy; for this disease, Delic and coworkers [52] demonstrated that the expression of urinary exosomal miRNAs in patients with microalbuminuria is different when compared with diabetic patients with normal levels of albuminuria, suggesting the possibility of predicting the course of the disease by analysing urinary miRNAs. These short non-coding RNAs could also play an important role in diagnosis and the follow-up of CD patients, since Felli and coworkers [53] identified three circulating miRNAs (miR-192-5p, miR-215-5p and miR-125b-5p) that were able to discriminate between CD patients at diagnosis, CD patients on a GFD and healthy controls. The adoption of these markers has been proposed for use in diagnosing CD in children exhibiting high TGA-IgA levels, but below 10 times the upper limit, who would usually undergo an esophagogastroduodenoscopy [54]. Elevated levels of these miRNAs in the blood, even before CD diagnosis, also indicate their potential as disease predictors. An interesting study conducted by Tan and collaborators [55] evaluated 53 CD-associated miRNAs from participants in the PreventCD study; eight of these fifty-three miRNAs were found to be elevated almost two years before the increase in TGA-IgA and returned to normal values during the GFD, while two of them (miR-150-3p and miR-150-5p) showed lower levels before the diagnosis and higher levels during the GFD, suggesting a potential role for miRNAs in the follow-up of CD. The cellular origin of the 53 miRNAs studied remains unknown, even if 15 of them are expressed in intestinal biopsies with mucosal damage from CD as well as in patients with inflammatory bowel disease (IBD) [56]. Based on this evidence, it was supposed that some miRNAs, such as miR-21, could be markers of intestinal inflammation, regardless of the disease, while others may be specific to CD.

The monitoring of GFDs remains an ongoing topic of discussion. One future challenge is to understand how to incorporate new monitoring techniques, particularly GIP and miRNA detection, into everyday clinical practice. Additionally, it is crucial to learn how to properly manage not only voluntary dietary lapses with enduring impacts but also occasional and unintentional deviations.

### 3.4. Gluten Contaminations

The European Codex Alimentarius [57] defines a product as gluten-free if it contains less than 20 ppm of gluten. However, studies show that the tolerance for gluten varies widely among individuals, with some being able to tolerate higher amounts (34–36 mg/day) [58,59] while others experience mucosal damage even with lower levels (less than 10 ppm). Despite careful preparation, the risk of cross-contamination is high, making strict adherence to a GFD challenging. Research has found that both labelled gluten-free products and naturally gluten-free foods can be contaminated, with a higher risk for the latter. Verma et al. [60] analysed 200 commercial products that are certified as gluten-free in Italian supermarkets: 87% were safe, with a gluten level <10 ppm, 4.5% contained gluten between 10 and 20 ppm and 9% were clearly contaminated with more than 20 ppm. Most of the contaminated foods contained oats, buckwheat and lentils. Another study, conducted in India on 794 products, evidenced that 10.8% of labelled GF products and 11.8% of non-labelled (naturally GF) products had a gluten content of >20 ppm. The most frequently contaminated products were flours, coarse grains, pasta and snack foods [61]. While the safety of GF food has improved over the years, studies report variable contamination rates (from 0.5% to 36%) depending on the study design and the analytical methods used [62,63]. Hidden sources of gluten, such as “vital gluten” found in several food products, medications and cosmetics, pose a danger to CD patients, emphasizing the need for vigilant label reading and an awareness of hidden gluten sources [64].

A special case which deserves to be mentioned is that of oats. The safety of oats in a GFD is still a matter of discussion. If pure, oats appear to be well tolerated in moderate amounts (20–25 g/day for children and 50–70 g/day for adults [65]) by most CD patients in remission, and harmful for less than 1%. In addition, oats are also a source of antioxidants, dietary fibre and unsaturated fatty acids, and that can make them a great ally in the GFD. The doubts about oats arise from the production line, which is the same as wheat, rye and barley, and which is often cause of oat cross-contamination with gluten-containing grains. Study design, and the type and purity of oats in different countries are the reasons why conducted studies about oat safety are still inconclusive [66,67]. Indeed, a study that analysed the gluten contamination of oats used in 12 important clinical studies showed how this contamination was related to the presence of symptoms in study patients, even if the estimated amount of gluten in the products did not seem to have an impact on the morphological outcome in treated patients [68]. Studies indicate a dose-dependent association between gluten intake and CD-relapse risk, ranging from 0.2% with 6 mg/day to 100% with 1.5 g/day of gluten [69]. However, to date, there are no consistent data that allow us to specifically quantify the impact that gluten contamination has on the course of a patient’s disease. A study conducted on 17 diet-adherent non-responsive celiac disease patients who underwent a Gluten Contamination Elimination Diet (GCED), consisting of whole unprocessed foods, showed that this diet was able to induce symptom resolution in 14 patients [70]. Another study, conducted in 2016 on asymptomatic patients who had not achieved mucosal recovery, showed that neither a GF diet nor a GCED diet were able to improve histologic parameters in these patients [14].

### 3.5. Nutritional Imbalances

While excluding gluten itself does not pose specific nutritional problems, the gluten-free diet often lacks essential vitamins, minerals and fibre [16]. Additionally, many gluten-free industrial products have poor nutritional quality and flavour and are expensive [14]. Concerns still exist regarding whether celiac-compliant patients following a gluten-free diet achieve a nutritional balance. These doubts arise from the fact that GF products are often rich in saturated fatty acids and cholesterol and have higher glycaemic indexes [16,71], with a consequent increase in cardiovascular risk and insulin resistance, weight gain and metabolic syndrome [72]. Typically, following a GFD, CD patients decrease complex carbohydrate consumption, due to the fear of unconscious gluten intake, and increase the consumption of simple sugars [73]. A study comparing GFD- adherent and non-adherent CD adolescents found that the adherent group had a significantly higher nutritional imbalance and a higher prevalence of overweight and obesity compared to healthy subjects and to the non-adherent group [74].

The amount of protein intake is still debated, with some studies asserting that CD patients have a greater intake of protein due to their habit of eating meat more frequently [75], while others demonstrate the contrary [76]. Moreover, CD patients tend to have an imbalanced intake of vitamins and minerals, with a highlighted low intake of micronutrients, minerals and fibre on a GFD [77]. Among micronutrients, vitamin D deficiency is particularly dangerous, because of the higher risk for CD patients of developing osteoporosis. Its supplementation is recommended, at least during the first year of a GFD, to compensate its intestinal malabsorption [78]. CD patients generally suffer from low levels of B vitamins caused by both malabsorption and their reduced presence within GF foods. This may cause increased homocysteine levels, whose prevalence is higher in CD patients after many years on a GFD [79]. Mineral deficiencies observed in CD patients seem to be caused by lower mineral contents in GF food than their gluten-containing analogues. A particular case is that of iron since, during a GFD, ferritin reserves need to be completely replaced thanks to mucosal healing. Gluten-free industrial products do not have a high iron content, so low iron levels are considered a common complication of CD and request particular attention, especially in women [80].

Overcoming this high risk of malnutrition is not a simple problem, and it is very important to educate patients on a healthy and balanced gluten-free regimen, also with the help of dietitians, and to monitor blood nutrient values through the years. Some helpful suggestions to improve the diet are to promote the consumption of naturally gluten-free food such as vegetables, fruits, legumes, nuts and pseudocereals, to carefully choose the source and amount of complex carbohydrates and proteins and to always prefer GF products fortified with micronutrients and vitamins [73].

### 3.6. Psychosocial Quality of Life in Patients on GFD

The strict nature of a GFD poses several challenges for patients, including limited food choices, difficulties in finding gluten-free options and the need for meticulous meal planning [15]. In a study conducted with a self-administered survey, including the standard quality of life (SF-QoL) questionnaire, CD patients reported a significant negative impact of the GFD on quality of life in social settings. Although this negative impact significantly diminished over time, it persisted in domains such as dining out and travelling. Notably, patients diagnosed with CD in childhood experienced a lesser impact on their quality of life as adults [81]. Patients following a gluten-free diet may experience psychological distress and anxiety related to their condition. The fear of accidental gluten exposure and its potential consequences can lead to heightened anxiety and hypervigilance. Studies focusing on psychological issues have revealed that individuals with celiac disease suffer from depression, anxiety, lower health-related quality of life and poorer overall psychological well-being that the general population. Furthermore, anxiety and depression are correlated with lower adherence to a GFD and poor adaptation to the disease condition [15]. Higher levels of anxiety were found particularly among CD women, who expressed more concern about the impact on socializing with friends and other important aspects of their life [82,83]. It cannot be doubted that a GFD can impact social interactions, leading to feelings of isolation and exclusion, especially in countries where gluten-containing foods are prevalent. CD patients also had a significantly higher prevalence of social phobia and depression compared to healthy subjects, with no significant differences between newly diagnosed subjects and patients on a GFD for a long time [84].

Several coping strategies and support systems can improve the psychosocial well-being of patients on a GFD. Educational programs, such as dietitian-delivered education, have shown positive effects in increasing knowledge, confidence and overall satisfaction with the diet. A prospective study on 53 newly diagnosed adult CD patients employed the Short Form 36 Health Survey, the Gastrointestinal Symptoms Rating Scale and the Beck Depression Inventory at diagnosis, one year and beyond four years of treatment. At one year, a significant improvement in quality-of-life indicators was observed, with scores comparable to healthy subjects. However, at four years, the Short Form 36 Health Survey scores and Beck Depression Inventory score showed significant deterioration compared to the one-year mark. In turn, long-term impairment of quality of life was also associated with the deterioration of compliance to the GFD [85]. Social media, with support groups and online communities, provide new opportunities for patients to connect, share experiences and receive emotional support [86].

Overall, the psychosocial quality of life of patients on a GFD can be significantly affected by the dietary restrictions. Healthcare professionals should address these challenges and provide support through education programs, support networks and psychological interventions. By addressing the psychosocial aspects, patients can better cope with the demands of a gluten-free lifestyle and improve their overall well-being [38].

### 3.7. Current and Future Perspectives on GFDs

#### 3.7.1. New Ingredients for New Gluten-Free Products

Wheat flour is a crucial component of the Mediterranean diet. Gluten plays a pivotal role in providing the desired texture of dough, including softness, elasticity and cohesion. Additionally, wheat flour is rich in fibres, minerals, B vitamins, selenium and other minerals. Proteins with properties similar to those of gluten (prolamins) can be found in other cereal species, such as hordeins in barley, secalins in rye and avenins in oats [33].

Nevertheless, in the absence of gluten, preparing high-quality gluten-free food can be challenging. Gluten-free flours, such as rice, pseudocereals, maize, millet, sorghum, chestnut, chia flours and legumes, offer good rheological and nutritional properties and taste [33,87], but often other ingredients have to be added to achieve a comparable high-quality gluten-free dough [43,88].

Also, starch plays a key role in breadmaking, because of its ability to gelatinize during the process; the elimination of wheat flour can therefore compromise the quality of the leavened dough. To overcome this problem, new types of gluten-free wheat starches have been created, such as hydroxypropylated, acetylated and cross-linked starches, that have water-binding properties and enhance dough volume. Another possibility is the addition to the dough of gluten-free starches from cassava, potatoes, tapioca, beans, corn and rice [33,88].

The addition of dietary fibres to gluten-free flours helps to confer water-binding and gel-forming properties to the dough, improving the loaf’s volume, and may compensate for the fibre deficiency typical of a GFD. Various types of fibre, such as β-glucan, inulin, apple pomace, oligofructose, bamboo fibre and carob fibre, legume, rice and cereal bran can be incorporated. Particularly, inulin has been demonstrated to reduce dough viscosity and increase the gelatinization temperature [33,88].

Hydrocolloids like guar gum and xanthan gum serve as excellent substitutes for gluten due to their ability to form polymeric structures, bind water and form films and networks which enhance the texture of gluten-free products without any flavour alteration. Other excellent types of hydrocolloids are HPMC (hydroxypropyl methyl cellulose) and CMC (carboxymethyl cellulose). HPMC has the ability to reduce cholesterol, increase loaf volume and retain gas, while CMC increases the porosity of the dough and contributes to the overall appreciation of the gluten-free products in terms of taste, consistency, uniformity and crunchiness [33,88]. Also, food-processing technology may improve dough structure in GF products. For example, the use of transglutaminase and the optimization of fermentation and baking processes may greatly improve the overall sensory quality of gluten-free bread [89].

Because of the absence of gluten, a GFD could become lower in proteins compared to gluten-containing diets. The addition of some proteins, like egg albumin, legumes, rice bran, soy, pea and lupine may improve the taste and rheological quality of gluten-free food, and it is also considered generally healthier than gluten, which is poor in essential amino acids. Enzymes (usually endopeptidase) in free form or bound to hydrogels for protection against stomach digestion, able to detoxify the gliadin peptide, could be added to food or supplements to mitigate the adverse effects of gluten exposure. While research in this area is ongoing, hydrogel-encapsulated enzymes hold potential as a novel approach to enhance the quality of life of CD patients [90].

Sourdough fermentation obtained using lactic acid bacteria is another promising strategy which can have a double benefit on gluten-free products. First of all, it improves the rheological properties and the structure of dough, increasing the volume, softness and final elasticity of the gluten-free bread; on the other side, it minimizes the immunological response, hydrolysing alfa-gliadin fragments and reducing the level of gluten, often to under 10 ppm [91].

Polyphenols are a heterogeneous group of natural molecules from the plant kingdom endowed with many biological activities, including antioxidant, anti-inflammatory, anti-allergic and anti-microbial, and protection against cancer and cardiovascular diseases [92]. A controversial effect of polyphenols, usually interpreted as a side effect, is their antinutritive property, due to the inhibition of digestive enzymes and the consequent reduction in nutrient absorption. The utility of these molecules in CD is due to their ability to interact with proteins rich in glutamine and proline through covalent or non-covalent bonds, avoiding the downstream catabolism leading to the so-called gluten toxic peptides. For this purpose, those most effective in binding gluten proteins are flavonoids, anthocyanidins, flavan-3-ols and flavonols. The beneficial action of polyphenols in celiac disease occurs in a complex mechanism: on the one hand, polyphenols create a steric clutter that, associated with an inhibition of digestive enzymes, reduces gluten absorption; on the other hand, as shown in previous studies [93], it appears that flavonoids may increase tight junctions between cells of the intestinal epithelium, making it more difficult for gluten peptides to reach the lamina propria.

Novel gluten-free wheat flours have emerged as a promising solution, showing superior nutritional profiles in comparison to conventional gluten-free options [94,95,96]. These flours frequently boast an elevated protein content, optimized amino acid profiles and heightened levels of dietary fibre. Specialized processing techniques, such as genetic modification, enzymatic treatments and deamination processes [97,98], are harnessed to diminish immune reactivity while safeguarding the essential functional attributes of wheat.

Alternative pseudocereal flours like amaranth, buckwheat, chia and quinoa have also gained prominence. Pseudocereal grains, devoid of gluten proteins, offer an abundance of high-quality proteins, cholesterol-lowering glycaemic control and free-fatty-acid-reducing carbohydrates [99,100,101]. Pseudocereal proteins exhibit no immune toxicity for celiac patients, highlighting their safety [102,103,104]. The inclusion of amaranth and quinoa flour in gluten-free bread formulations has demonstrated a minimal impact on texture and volume, resulting in bread loaves that achieve a ‘moderately acceptable’ rating in sensory evaluations, thereby assuring their incorporation without compromising overall quality contributing to the production of acceptable gluten-free bread [105,106]. Furthermore, quinoa and amaranth have been used in gluten-free cake formulations at different concentrations (from 0% to 30%) resulting in palatable and healthier products [107].

Legume and chestnut flours have been successfully integrated into gluten-free bread to improve texture, starch integrity and fibre content [81]. The promising synergy between chickpea protein and tiger nut lipids offers an innovative approach to preserving the fundamental baking characteristics of bread loaves, circumventing the need for conventional emulsifiers and shortenings. This novel amalgamation not only sustains bread volume and crumb integrity but also culminates in a sensory experience that aligns with acceptability criteria [108]. Chickpea flour, renowned for its high protein and fibre content, has emerged as a promising ingredient for GFDs. Interestingly, the overall acceptability levels of chickpea flour cookies were quite high [109]. Buckwheat and chia flours introduce enhancements in moisture retention and interactions among matrix biopolymers, thereby enhancing breadmaking performance, particularly in commercial settings. Also, green plantain flour and carob germ flour emerge as noteworthy substitutes for wheat flour, yielding a viscoelastic dough and a high-quality gluten-free bread, albeit with a minor reduction in loaf volume [110,111,112].

Some studies suggest that these flours may elicit a reduced immune response compared to traditional wheat flours [98]. However, individual responses may vary, and caution should be exercised when introducing new gluten-free wheat flours into the diet. Some gluten-free flours, such as corn and rice flour, have a high glycaemic index [100], which may increase the risk of metabolic syndromes in CD patients [113]. Moreover, in a recent study, gluten-free cracker-type snacks were created to offer a balanced supply of essential amino acids, a lower glycaemic index and reduced caloric intake, while maintaining sensory appeal [114].

Research has also focused on ancient low-gluten wheat varieties [55] that could be suitable for CD patients, avoiding the activation of the immune system. In vitro studies have suggested *Triticum monococcum* spp. Monococcum gluten as having poor immunogenicity; however, comparing two monococcum lines, Monlis and ID3311, it has been demonstrated [98] that both can activate the T lymphocyte even if ID3311 cannot activate innate immunity, becoming a potential tool for preventing CD.

Despite their potential benefits, several limitations exist regarding the safety of new gluten-free wheat flours. The limited availability of these flours, the potential cross-reactivity with gluten and the need for further research and standardization are important considerations.

Moreover, although pseudocereals or gluten-free cereals used for the production of these flours certainly have a good nutritional profile, the need to add additives to improve the structural and organoleptic characteristics of the final products makes them processed foods (NOVA 4 classification) in all respects [115]. Thickeners such as xantan gum, guam gum, methyl cellulose and emulsifiers such as DATEM (diacetyl tartaric ester of mono- and diglycerides) and SSL (Sodium Stearoyl lactate) are just a few of the many additives used to produce GF bread, pasta or bakery products [88]. There is rising evidence that such food additives may contribute to dysbiosis, intestinal barrier disruption and inflammation in the gut [116,117]. Since these foods are designed for daily consumption, long-term impact-safety studies, larger clinical trials and post-market surveillance are essential to assess the safety and efficacy of these flours in the context of gluten-related disorders. Table 2 summarizes the advantages and disadvantages of the most common gluten-free flours.

#### 3.7.2. New Therapies on the Horizon beyond the Gluten-Free Diet

It is widely acknowledged that a GFD is not embraced by some patients. Consequently, an increasing number of studies are focusing on pharmacological treatment that targets pivotal elements in the pathogenesis of the disease. The most promising approaches involve gluten neutralisation and TG2 inhibition and immunomodulation. Noteworthy gluten neutralization agents [135] include: latiglutenase (ALV003, Alvine Pharmaceuticals Inc., San Carlos, CA, USA) [136], a mixture of endopeptidases that proteolyze gluten and reduce its immunogenicity; TAK-062 (Takeda Pharmaceutical Co. Ltd., Osaka, Japan) [137,138], an endopeptidase designed to target proline and glutamine peptides; and BL-7010 (BioLineRx Ltd., Modi’in-Maccabim-Re’ut, Israel) [136], a synthetic polymer that binds to gliadin peptides, facilitating their elimination through the gastrointestinal tract. The main targets of gluten transport centre on tight junction proteins, particularly zonulin. Larazotide acetate (AT-1001, Alba Therapeutics Corp., Baltimore, MD, USA) [139] serves as a tight junction regulator, reducing intestinal permeability and, consequently, the amount of gluten reaching the lamina propria. Recent clinical trials are also exploring the efficacy and safety of an intestinal TG2 inhibitor (ZED1227) (Dr. Falk Pharma GmbH, Freiburg im Breisgau, Germany) [140], which binds to the active site of TG2, leading to a partial interruption of the cascade of events responsible for immune activation. Furthermore, immunotherapeutic agents hold significant promise. The most attractive results have been obtained from studies involving NexVax2 (ImmusanT Inc., Cambridge, MA, USA) [141], a tolerance-inducing vaccine designed to desensitise patients to gluten. This vaccine could be invaluable in cases of accidental gluten ingestion. Nanoparticles [142], synthetic drugs with diverse mechanisms for action and multiple targets, have also shown promise. For instance, some nanoparticles containing siRNAs (small interfering RNAs) are capable of silencing TG2 or specific interleukin genes. Additionally, HLA DQ2/DQ8 (DONQ52, Chugai Pharmaceutical Co. Ltd., Tokyo, Japan/ALDOMET (Methyldopa), Allphamed PHARBIL Arzneimittel Ltd., Göttingen, Germany) blockers [143] have gained attention for their ability to bind the active site of HLA, thereby disrupting immune activation.

Microbiota and its correlations with immune functions have been considered as a potential therapeutic target in CD patients. Various studies have assessed the therapeutic potential of probiotics and postbiotics in active CD patients, and some have achieved positive results, with a shift of the microbiota towards eubiosis associated with a reduction in some circulating inflammatory cytokines such as TNF-a, an improvement in intestinal permeability or a reduction in gastrointestinal symptoms [143]. Microbial-based therapies offer CD patients new therapeutic perspectives, even though a GFD comes before any other therapeutic modalities.

## 4. Conclusions

A GFD remains the primary treatment for CD, and European legislation requires schools, hospitals and public restaurants to provide gluten-free meals. Despite the proven efficacy of a GFD and the increased availability of gluten-free meals, adhering to the GFD is not always straightforward and can entail various complexities and hazards. Main challenges faced daily by CD patients are inadvertent gluten exposure, high costs and the poor quality of gluten-free food. Researchers are actively focusing on these issues, endeavouring to develop alternative approaches and innovative production techniques. These endeavours encompass the creation of safe gluten-free wheat flours, the exploration of sourdough fermentation, and the incorporation of hydrocolloids, enzymes, starches and dietary fibres in industrial gluten-free foods to enhance the overall quality, flavour and safety of these products.

The management of the disease and all these daily obstacles can lead to a poor quality of life and psychosocial distress, especially in young people. Despite the pivotal role of the diet in their life, nutritional imbalances, mostly vitamin and mineral deficiencies, are frequent in CD patients, and the risk of obesity, metabolic syndrome and cardiovascular diseases is higher. Nonetheless, strict adherence to the GFD remains imperative, warranting vigilant supervision by attending physicians. Multiple methods are available for monitoring dietary compliance, although none have been universally deemed entirely effective. Alongside conventional approaches such as clinical assessment, dietary questionnaires, serology and endoscopic evaluation, the detection of GIPs in faecal and urinary samples holds promise as a non-invasive method to assess gluten ingestion. However, additional research is needed to fully understand its correlation with other markers and mucosal damage. In this setting, emerging diagnostic tools such as urinary miRNAs hold potential for the diagnosis and follow-up of gluten-related disorders.

Overall, a comprehensive understanding of gluten-related disorders, the challenges and risks of a GFD, and also the adequate monitoring of adherence can contribute to improved therapeutic management and an increase in the quality of life for affected individuals. In conclusion, this narrative review prompts readers to contemplate the potential for further improvement in the management of CD. It particularly emphasizes the need for advancements in various aspects, including the quality of gluten-free industrial products, the development of novel alternative flours, the development of drug therapies and the implementation of robust methods to monitor adherence and the possible consequences of a GFD.

## Figures and Tables

**Table 1:** Overview of main advantages and disadvantages of GFD-monitoring methods used today in clinical practice.

	Advantages	Disadvantages
Clinical Assessment	Non-invasive Rapid Cheap	Low sensitivity and specificity
Validated questionnaires	Non-invasive Rapid Cheap Good information about patient’s diet	Subjective Poor correlation with symptoms and histological findings
Serology	High sensitivity and specificity	Different available testing platform Poor correlation with symptoms and histological findings Unable to detect occasional transgression
Biopsies	High sensitivity and specificity	Invasive Expensive Unsuitable for frequent monitoring
GIP Detection	Non-invasive Rapid Cheap Able to detect occasional transgression	Not yet clear correlation with mucosal damages and serology

**Table 2:** Advantages and disadvantages of the most common gluten-free flours.

Flour Type	Advantages	Disadvantages	Ref.
Almond flour	Rich in protein and dietary fibre Good sensory properties Cholesterol-lowering effect	Can be dense High in calories	[118]
Coconut flour	High fibre content Mild sweetness	Absorbs a lot of moisture and requires more liquid	[119]
Rice flour	Neutral flavour Digestibility Hypoallergenic proteins	May result in a gritty texture if not finely ground	[120]
Tapioca flour (from the cassava root)	Starchy flour used as thickener Light texture Neutral taste	Lacks significant nutritional value compared to other flours	[121]
Chickpea flour	High protein content High water retention capacity Good acceptability	Strong flavour	[109]
Quinoa flour	Excellent protein source Mild flavour Increased loaf volume More uniform crumb structure	Can be expensive compared to other flours	[103]
Buckwheat flour	High nutritional value Antioxidant activity Reduced glycaemic index	Strong, bitter flavour	[122,123]
Sorghum flour	Neutral taste Good texture	May require additional binders	[124]
Millet flour	Mild flavour Good texture High contents of antioxidants and health-promoting polyphenols	May require blending with other flours	[125]
Amaranth and quinoa flour	High in fat, fibre content and complete protein source Minimal impact on texture and volume	Can have a strong flavour	[107]
Cassava flour	Starchy and neutral taste High nutritional value Good texture	May result in denser baked goods	[126,127]
Soy flour	Rich in protein Good texture Increased binding properties	Strong soy taste can be overpowering	[128]
Potato flour	Provides moisture and a soft texture in baked foods High fibre content	Limited nutritional value and flavour	[129]
Teff flour	Mild flavour High nutritional value and complete protein source Low glycaemic index	Not widely available	[130]
Green Banana flour	High in resistant starch for gut health Low glycaemic index	Limited flavour and may require recipe adjustments.	[131]
Chestnut flour	Sweet flavour High nutritional value and antioxidant properties	Limited availability and can be expensive	[132]
Sesame flour	Rich in healthy fats and protein Antioxidant properties. Beneficial effect on gut microbiota Good sensorial acceptance	Modified surface appearance (colour and cracking)	[133]
Hazelnut flour	High nutritional value and health benefits Good sensory characteristics	Low water absorption of the flour Increased dough development time Reduced dough stability during kneading	[134]
Green plantain flour	Its incorporation in a flour blend of rice flour and GF wheat starch showed good potential for improving the quality of GF bread		[110]
Chia flour	If added to rice flour, the reduction in loaf volume, crumb firmness and crumb moisture is negligible	Chia flour alone is not suitable for bread production	[111]
Carob germ proteins	Good viscoelastic properties, high nutritional value	Not widely available	[112]

## Data Availability

Data are available in a publicly accessible repository. The data presented in this study are openly available in the PubMed database.

## Abbreviations

CD: celiac disease; GFD: gluten-free diet; NCGS: non-celiac gluten sensitivity; WA: wheat allergy; CDAT: Celiac Dietary Adherence Test; GIP: Gluten Immunogenic Peptide; HIIT: High-Intensity Interval Training; SDE: Standardized Dietician Evaluation.
